# How the Cobra Got Its Flesh-Eating Venom: Cytotoxicity as a Defensive Innovation and Its Co-Evolution with Hooding, Aposematic Marking, and Spitting

**DOI:** 10.3390/toxins9030103

**Published:** 2017-03-13

**Authors:** Nadya Panagides, Timothy N.W. Jackson, Maria P. Ikonomopoulou, Kevin Arbuckle, Rudolf Pretzler, Daryl C. Yang, Syed A. Ali, Ivan Koludarov, James Dobson, Brittany Sanker, Angelique Asselin, Renan C. Santana, Iwan Hendrikx, Harold van der Ploeg, Jeremie Tai-A-Pin, Romilly van den Bergh, Harald M.I. Kerkkamp, Freek J. Vonk, Arno Naude, Morné A. Strydom, Louis Jacobsz, Nathan Dunstan, Marc Jaeger, Wayne C. Hodgson, John Miles, Bryan G. Fry

**Affiliations:** 1 Venom Evolution Lab, School of Biological Sciences, University of Queensland, St. Lucia, QLD 4072, Australia; nadya.panagides@gmail.com (N.P.); tnwjackson@gmail.com (T.N.W.J.); rp1990@gmx.at (R.P.); dr.syedabidali@gmail.com (S.A.A.); jcoludar@gmail.com (I.K.); james.dobson@uqconnect.edu.au (J.D.); brittany.sanker@uq.net.au (B.S.); angelique.asselin@uq.net.au (A.A.); renancassant@gmail.com (R.C.S.); iwanhx@yahoo.com (I.H.); 2 QIMR Berghofer Institute of Medical Research, Herston, QLD 4049, Australia; maria.ikonomopoulou@qimrberghofer.edu.au (M.P.I.); John.Miles@qimrberghofer.edu.au (J.M.); 3 School of Medicine, The University of Queensland, Herston, QLD 4002, Australia; 4 Department of Biosciences, College of Science, Swansea University, Swansea SA2 8PP, UK; kevin.arbuckle@swansea.ac.uk; 5 Monash Venom Group, Department of Pharmacology, Monash University, Clayton VIC 3800, Australia; daryl.yang@monash.edu (D.C.Y.); wayne.hodgson@monash.edu (W.C.H.); 6 HEJ Research Institute of Chemistry, International Centre for Chemical and Biological Sciences (ICCBS), University of Karachi, Karachi 75270, Pakistan; 7 Working Group Adder Research Netherlands, RAVON, 6525 ED Nijmegen, The Netherlands; info@eyecreations.nl; 8 Working Group Venomous Bites Netherlands, RAVON, 6525 ED Nijmegen, The Netherlands; jeremie@ratelslangen.nl; 9 Naturalis Biodiversity Center, 2333 CR Leiden, The Netherlands; rovdbergh@me.com (R.v.d.B.); freek.vonk@naturalis.nl (F.J.V.); 10 Institute of Biology Leiden (IBL), Leiden University, Sylviusweg 72, 2333 BE Leiden, The Netherlands; h.m.i.kerkkamp@biology.leidenuniv.nl; 11 Snakebite Assist, Pretoria ZA-0001, South Africa; afnaude@worldonline.co.za; 12 Department Pharmacology, University of Pretoria, Pretoria ZA-0001, South Africa; morne.strydom@synexus.com; 13 SYNEXUS Clinical Research SA Pty Ltd., Pretoria ZA-0001, South Africa; 14 Zoology Department, University of Pretoria, Pretoria ZA-0001, South Africa; louissnakes@gmail.com; 15 Venom Supplies, Tanunda, South Australia 5352, Australia; nathan@venomsupplies.com; 16 Planet Exotica, 5 Avenue des Fleurs de la Paix, 17204 Royan, France; marc.jaeger@bluewin.ch; 17 Australian Institute of Tropical Health and Medicine, James Cook University, Cairns, QLD 4878, Australia

**Keywords:** cytotoxin, cobra, *Hemachatus*, *Naja*, *Ophiophagus*, Elapidae, evolution, antipredator defense

## Abstract

The cytotoxicity of the venom of 25 species of Old World elapid snake was tested and compared with the morphological and behavioural adaptations of hooding and spitting. We determined that, contrary to previous assumptions, the venoms of spitting species are not consistently more cytotoxic than those of closely related non-spitting species. While this correlation between spitting and non-spitting was found among African cobras, it was not present among Asian cobras. On the other hand, a consistent positive correlation was observed between cytotoxicity and utilisation of the defensive hooding display that cobras are famous for. Hooding and spitting are widely regarded as defensive adaptations, but it has hitherto been uncertain whether cytotoxicity serves a defensive purpose or is somehow useful in prey subjugation. The results of this study suggest that cytotoxicity evolved primarily as a defensive innovation and that it has co-evolved twice alongside hooding behavior: once in the *Hemachatus + Naja* and again independently in the king cobras (*Ophiophagus*). There was a significant increase of cytotoxicity in the Asian *Naja* linked to the evolution of bold aposematic hood markings, reinforcing the link between hooding and the evolution of defensive cytotoxic venoms. In parallel, lineages with increased cytotoxicity but lacking bold hood patterns evolved aposematic markers in the form of high contrast body banding. The results also indicate that, secondary to the evolution of venom rich in cytotoxins, spitting has evolved three times independently: once within the African *Naja*, once within the Asian *Naja*, and once in the *Hemachatus* genus. The evolution of cytotoxic venom thus appears to facilitate the evolution of defensive spitting behaviour. In contrast, a secondary loss of cytotoxicity and reduction of the hood occurred in the water cobra *Naja annulata*, which possesses streamlined neurotoxic venom similar to that of other aquatic elapid snakes (e.g., hydrophiine sea snakes). The results of this study make an important contribution to our growing understanding of the selection pressures shaping the evolution of snake venom and its constituent toxins. The data also aid in elucidating the relationship between these selection pressures and the medical impact of human snakebite in the developing world, as cytotoxic cobras cause considerable morbidity including loss-of-function injuries that result in economic and social burdens in the tropics of Asia and sub-Saharan Africa.

## 1. Introduction

Members of the front-fanged venomous snake family Elapidae are present throughout the tropical and temperate parts of the Old and New World. These snakes are responsible for many clinically significant bites to humans including fatalities. Species with potently cytotoxic venom, particularly members of the genus *Naja* (cobras) in Africa and tropical Asia, are major causes of loss-of-function injuries, which may render survivors unable to work [1,2,3]. Little reliable data exists for the incidence of morbidity following snakebite in developing countries, but it is predicted that the morbidity and mortality burden is considerable [4]. The permanent sequelae resulting from bites by cytotoxic species of snake can result in lifelong disabilities that render victims unable to perform manual labour. As snakebite is a disease that primarily affects the poor [5], and as young males are at highest risk [6,7], cytotoxic envenomations generate a considerable socioeconomic burden in developing countries. Understanding the evolutionary pressure that shapes the activity of snake venoms may aid attempts to address the snakebite crisis in sub-Saharan Africa and southern Asia both by providing valuable insights into the relationship of snake behaviour to venom activity and by assisting the development of effective and affordable snakebite therapeutics. Currently, the evolution of elapid snake venom, including its connection to behavioural and morphological patterns, is poorly understood. Specifically, little is known concerning the selection pressures that resulted in the evolution of potent cytotoxicity within the elapid snake family, particularly among cobras. Why snakes from a family typified by venoms rich in potent neurotoxins, which are devastatingly effective in prey subjugation [8,9], should evolve less potent cytotoxins and why this should have occurred primarily in one clade (cobras and their close relatives), are intriguing and important questions for evolutionary toxinology to address.

Elapidae is a large and diverse family which underwent a major radiation at the end of the Eocene period [10], spreading over much of Africa and southern Asia and ultimately reaching Australasia approximately 25 million years ago [11]. These fast moving snakes typically rely on cryptic colouration to remain undetected by predators during periods of rest. However, some lineages (*Naja* and closely related genera) have evolved defensive behaviour characterised by the extension of “hoods”, formed when elongate ribs in the neck are spread, which often reveal bright colours and intricate patterns [12]. This morphological and behavioural adaptation also calls for a “plan B”—a defensive strategy in case the display fails [13,14]. Typically, plan B involves painful defensive bites, but the ability to spit venom is believed, on the basis of variations in morphology and associated behaviour, to have evolved three times independently within the *Naja* + *Hemachatus* clade [13,15].

Most prior research has focussed on the spitting behavior itself, treating it as a peculiar oddity in nature without investigating associated evolutionary trends (e.g., venom composition) [16], and it has been described at the morphological [17,18], mechanical [12,19,20] and behavioural levels [13,21]. Hooding is a distinctive characteristic of the genera *Hemachatus*, *Naja* and *Ophiophagus* and members of these genera are found in Africa and Asia [22]. Since *Ophiophagus* is not closely related to *Naja* and *Hemachatus* [15,17], hooding has likely evolved on at least two separate occasions. Previous studies have investigated the morpho-kinetic action of hooding [16] or simply observed that hooding is a form of defensive reaction [12] that presumably functions as an aposematic signal or intimidation display by giving the appearance of being larger.

Myriad toxins are responsible for the cytotoxicity (cell death) of snake venoms. The tissue destruction that often results from envenomation by *Naja* spp. is caused by a specialised class of 3-finger toxins (3FTx)—the cytotoxins or “cardiotoxins” [1]. In *Ophiophagus* envenomations, on the other hand, L-amino acid oxidase is responsible [23]. Despite the iconic nature of the species involved, the connection between the hooding and spitting behaviours and the cytotoxicity of the venoms has not been previously investigated. Indeed, hooding behaviour has puzzled scientists for more than 200 years [14,24]. Thus, this project aimed to test the relationship between these three character states with a view to understanding how this suite of defensive adaptations has coevolved.

## 2. Results and Discussion

**Figure 1:**
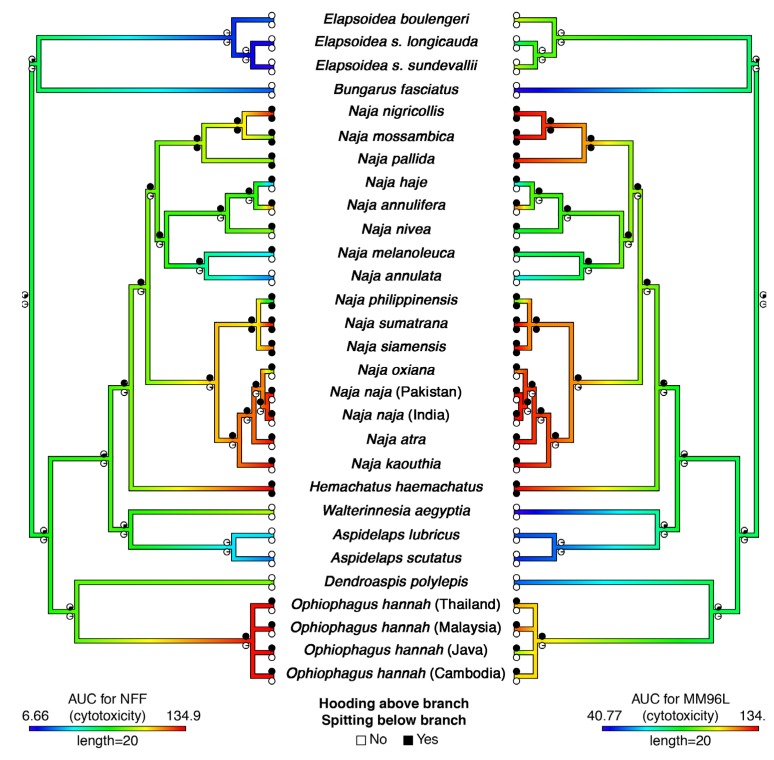
Ancestral state reconstructions of hooding, spitting, and cytotoxicity; based on ambiguous *Aspidelaps* species coded as non-hooding as well as the ambiguous *N. atra* and *N. kaouthia* also coded as non-spitting. Reconstruction over branches represents the AUC (area under the curve) for the non-transformed (NFF) cell line (left) and the melanoma (MM96L) cancer cell line (right), where warmer colours represent higher cytotoxicity against cell lines (raw data in Supplementary Tables). Pie charts are the same on both trees and represent estimates of ancestral states for hooding (above branch) and spitting (below branch) where black and white represent the trait being present or absent respectively. States at tips represent the data collected. Phylogeny follows Lee (2016) [11].

**Figure 2:**
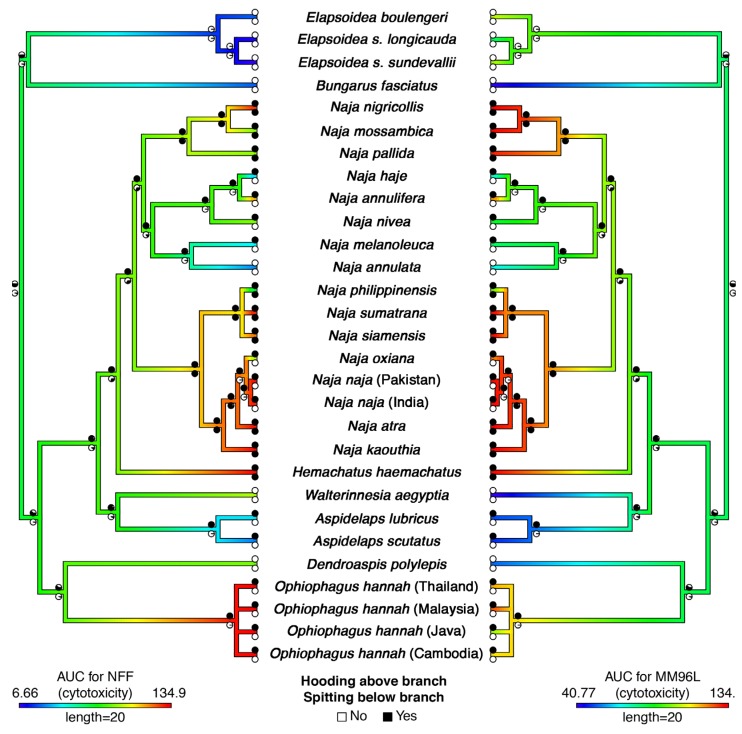
Ancestral state reconstructions of hooding, spitting, and cytotoxicity; based on ambiguous *Aspidelaps* species coded as hooding as well as the ambiguous spitters *N. atra* and *N. kaouthia* also coded as spitting. Reconstruction over branches represents the AUC for the non-transformed NFF cell line (left) and the melanoma (MM96L) cancer line (right) where warmer colours represent higher cytotoxicity against that cell line (raw data in Supplementary Tables). Pie charts are the same on both trees and represent estimates of ancestral states for hooding (above branch) and spitting (below branch) where black and white represent the trait being present or absent respectively. States at tips represent the data collected. Phylogeny follows Lee (2016) [11].

In order to account for variance by cell type, we tested the cytotoxic effects of crude venoms on one healthy-type cell line and one cancerous cell line and looked for congruence in effect between the two cell types. Colorimetric MTT testing revealed substantial variation between non-hooding and hooding species in relative toxicities across cell types and concentrations (Figure 1 and Supplementary Tables) (raw data in Supplementary Tables). Cytotoxicity of *Naja* venom appears to have increased on several separate occasions (Figure 1 and Supplementary Tables). Increased cytotoxicity was also observed in *Hemachatus* and *Ophiophagus*. Notably, increased cytotoxicity is not estimated to have occurred concurrently with hooding, but rather to have evolved subsequent to the evolution of hooding behavior (Figure 1 and Supplementary Tables). Exceptional hooding behavior itself is estimated to have evolved twice, once on the branch leading to *Ophiophagus* and once at the base of the clade *Naja* + *Hemachatus* (Figure 1). However, if weakly hooding species such as *Aspidelaps* are classified as ‘hooding’, we find modest evidence for an earlier single origin of hooding ability in clade comprising *Aspidelaps*, *Hemachatus*, *Naja* and *Walterinnesia* (Figure 2). According to this interpretation, *Walterinnesia* would have lost the propensity to hood, presumably as a result of their occupation of a nocturnal niche in which visual defensive displays are less effective.

Based on our ancestral state reconstructions, we found evidence that spitting behavior has evolved on three occasions (Figure 1), as predicted and based on morphological and behavioural adaptations [13,15]. To our knowledge, this is the first attempt to formally estimate the number of origins of spitting and confirms earlier suggestions by Wuster et al. (2007): one origin in *Hemachatus*, one in Asian *Naja*, and one in African *Naja* [25]. We found a difference in the placement of origins of spitting behavior in the Asian *Naja* clade based on how *N. atra* and *N. kaouthia* are coded (Figure 1 and Figure 2). These species are not considered ‘true’ spitters as they do not possess specialised morphological adaptations and do not commonly spit, however, both have been recorded to spit venom at a substantial distance on rare occasions [26,27]. When focused only on the highly specialised spitters, we find that spitting evolved at the base of the lineage of highly adapted Asian *Naja* (represented in this study by *N. philippinensis*, *N. siamensis* and *N. sumatrana*) (Figure 1). Alternatively, when including the weakly adapted cases (Figure 2), we find an origin of spitting at the base of the Asian *Naja* clade followed by a loss in the *N. naja* + *N. oxiana* clade Although counterintuitive at first glance, this scenario would interpret the limited spitting ability of *N. atra* and *N. kaouthia* as a vestigial relic of spitting ancestors and would also explain the increased cytotoxicity across the whole Asian *Naja* clade (Figure 2). Specialised morphological adaptations for spitting may also be a more recent innovation in the Asian *Naja* than in their African counterparts, as they are known to be less accurate spitters and the trait seems less fixed given the (at least) two species (*N. atra* and *N. kaouthia*) that contain both spitting and non-spitting individuals [28]. Nevertheless, more complete data regarding spitting behavior in these “quasi-spitters” would be required to rigorously verify this suggested scenario. Taken together, however, the evidence uncovered in the present study suggests that the evolution of hooding and defensive cytotoxic venom in turn produced the selection pressures that facilitated the evolution of spitting.

**Figure 3:**
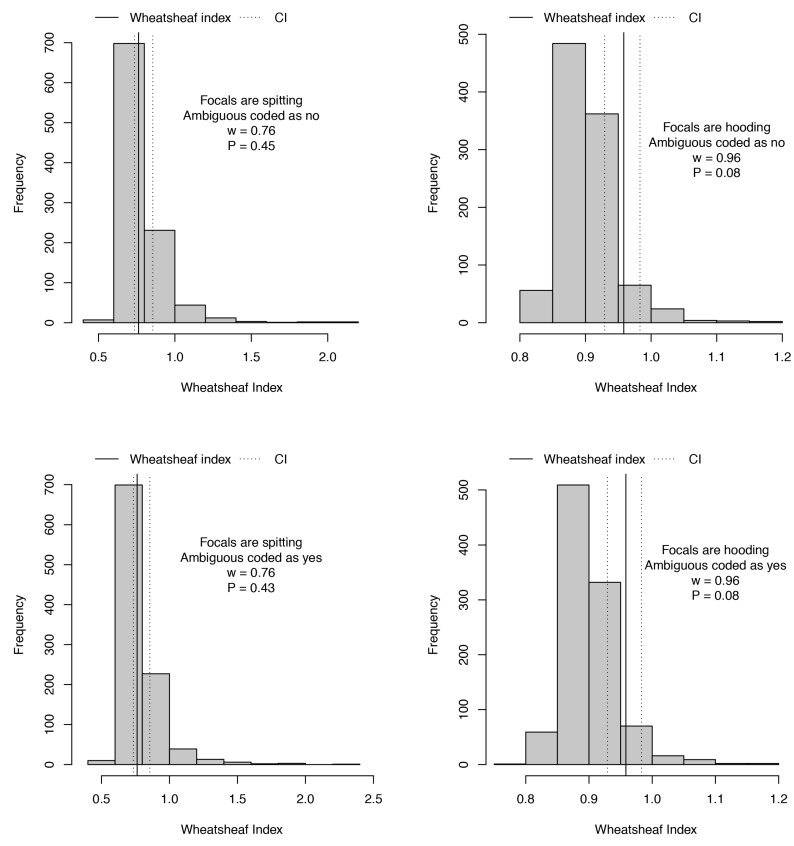
Analyses of strength of convergence in cytotoxicity in association with spitting or hooding behaviour, under both coding schemes for ambiguous species. Histograms show that the null distribution represented expected strengths of convergence in each analysis, with solid and dotted lines overlaid to show calculated Wheatsheaf index (w) and its 95% confidence interval respectively. Wheatsheaf index is presented alongside the *p*-value for exceptionally strong convergence in each case. Note that alternative codings make a little difference to results but convergence in cytotoxicity is slightly stronger (with a higher Wheatsheaf index) when associated with hooding than spitting behaviour.

In further statistical investigations of the relative importance of hooding versus spitting in the evolution of cytotoxicity, we found that the best supported pGLS model for toxicity to both NFF and MM96L cell lines included hooding but not spitting (NFF line, ambiguous coded no: t_1,27_ = 3.80, *p* = 0.0008; MM96L line, ambiguous coded no: t_1,27_ = 4.70, *p* = 0.0001; MM96L line, ambiguous coded yes: t_1,27_ = 2.63, *p* = 0.014). These models provide complementary and consistent support for hooding being more important than spitting in the evolution of cytotoxicity. Finally, we also applied the Wheatsheaf index [29], which controls for undue weight given to close relatives and also for the possible distributions of traits over a given tree, to ask whether convergence in cytotoxicity was stronger for spitting or hooding species. Although neither trait was significantly associated with exceptionally strong convergence across the tree (Figure 3), hooding was associated with stronger convergence in cytotoxicity than was spitting (*w*_spitting_ = 0.76, *p* = 0.44; *w*_hooding_ = 0.96, *p* = 0.08). Therefore, although it is difficult to make robust evolutionary inferences about suites of traits when each has very few origins, the consistent picture arising from the several statistical analyses utilised suggests that, in the evolution of this group of snakes, hooding is more strongly associated with cytotoxicity than spitting. Our results also display a clear link between the evolution of strong hood or body patterns, as part of enhanced visual displays, and parallel increases in cytoxicity.

**Figure 4:**
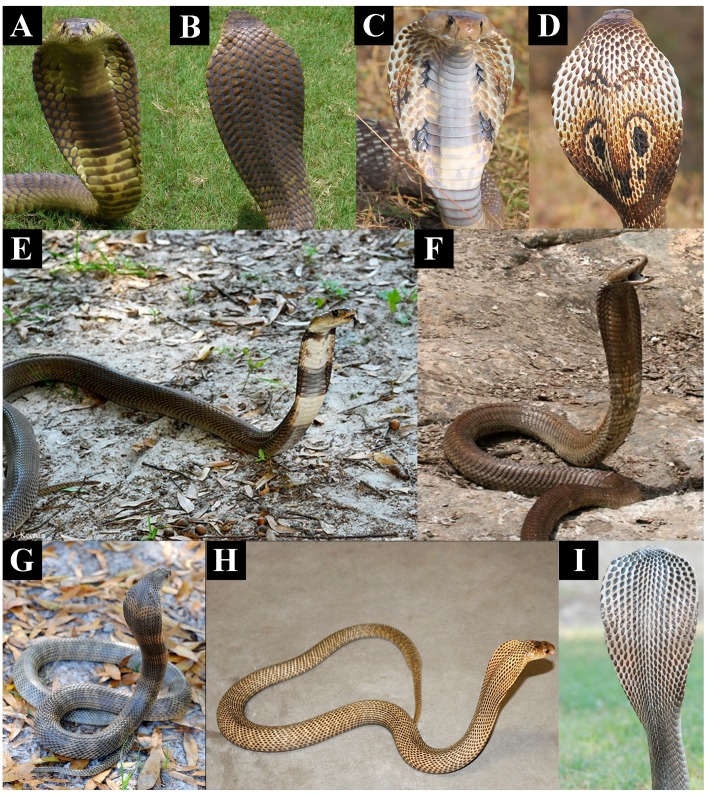
Relative degree of aposematic hood marking between (**A**,**B**) African (*Naja haje*) and (**C**,**D**) Asian (*Naja naja*) cobras with their higher levels of cytotoxicity;. Similar patterning to *N. haje* are seen in the the basally coloured African spitting cobras lacking aposematic marking like *N. haje* (**E**) *N. nubiae* and (**F**) *N. ashei.* Convergent reversal from aposematic markings to the basal drab coloured state accompanied by a lowering of cytotoxicity has occurred in (**G**) the Asian non-spitting cobra *N. oxiana* and (**H**) the spitting cobra *N. phillipinensis*. The Pakistan Sindh desert population of *N. naja* displays the aposematic hood marking as juveniles and subadults but not (**I**) as adults, without any loss of cytotoxicity. Photos: (**A**,**B**) Arno Naude; (**C**,**D**) Gowri Mallapur; (**E**) HG Hjim; (**F**) Anothony Childs; (**G**,**H**) Randy Ciuros; (**I**) Bryan Fry.

The most recent common ancestor of *Naja* and *Hemachatus* appears to have possessed moderately cytotoxic venom (Figure 1) and morphologically may have resembled today’s *Naja haje*, which is drab in colour, displaying little or no patterning when hooding (Figure 4A,B). These snakes strike nervously and flee when possible [30]. In both cell types tested the African non-spitting *Naja* such as *N. haje* were higher in cytotoxicity than non-hooding snakes but lower than all other *Naja*, especially those with aposematic markings (Figure 1 and Supplementary Tables). This pattern was consistent with that seen in another cytotoxicity studies which compared *N. haje* with *Naja mossambica* and *Naja nigricollis* [31]. The Asian non-spitting *Naja* have evolved broader hoods along with aposematic hood markings (Figure 4C,D) and are notorious for standing their ground when faced with a threat. These snakes were significantly more toxic to both cell types than African non-spitting *Naja* (Figure 1 and Supplementary Tables). Basal African spitting cobras such as *Naja nubiae* (Figure 4E) and *Naja ashei* (Figure 4F) resemble *N. haje* in being drably coloured with non-descript patterning, if any. Similar, possibly plesiomorphic, colour and patterning is seen in the non-spitting *Naja oxiana* and spitting *Naja philippinensis* Asian species, both of which are nested within clades of aposematically marked species. *N. oxiana* is sister to the ornate *N. naja* while *N. philippinensis* is related to the brightly banded species *N. siamensis*. In both cases, the non-aposematically marked state is accompanied by a decrease in cytotoxicity (Figure 1 and Supplementary Tables). It is notable that the desert dwelling population of *N. naja* from Pakistan, which becomes melanistic as adults (Figure 4I) but displays the aposematic hood markings as juveniles and subadults, does not show a decreased cytotoxicity relative to the aposematic hood marked population studied from India.

**Figure 5:**
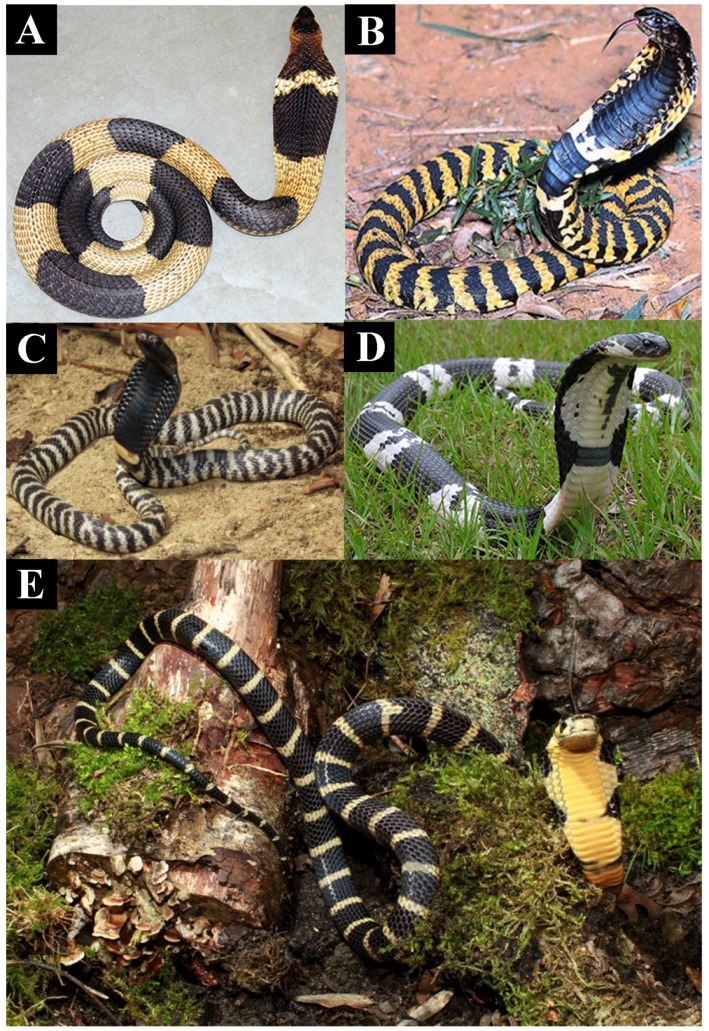
Convergent aposematic banding in the strongly cytotoxic species (**A**) *Naja annulifera*; (**B**) *Hemachatus haemachatus*; (**C**) *Naja nigricincta*; (**D**) *Naja siamensis*; and (**E**) *Ophiophagus hannah*; Photos by (**A**,**D**) Randy Ciuros; (**B**) Giuseppe Mazza; (**C**,**E**) Tom Charlton.

*N. annulifera* was the only African non-spitting cobra with an increase of cytotoxicity (Figure 1 and Supplementary Tables). This species is also unique amongst African non-spitting *Naja* in being brightly banded (Figure 5A). The evolution of aposematic body banding occurred convergently in four other lineages: *Hemachatus haemachatus* (Figure 5B), *N. nigricincta* (Figure 5C), *N. siamensis* (Figure 5D), and *O. hannah* (Figure 5E). In *N. siamensis* the hood markings are obscured by solid black colouring suggesting (at least for this species) an inverse relationship between aposematic hood and body markings—non-spitting Asian cobras often have aposematic hood marks (Figure 3D). Unlike African spitting cobras, the Asian spitting cobras do not display a dramatic rise in cytotoxicity to either cell type relative to aposematically marked non-spitting Asian *Naja*. However, as stated above, the aposematic *N. siamensis* displayed higher toxicity than *N. philippinensis* (Figure 1 and Supplementary Tables).

**Figure 6:**
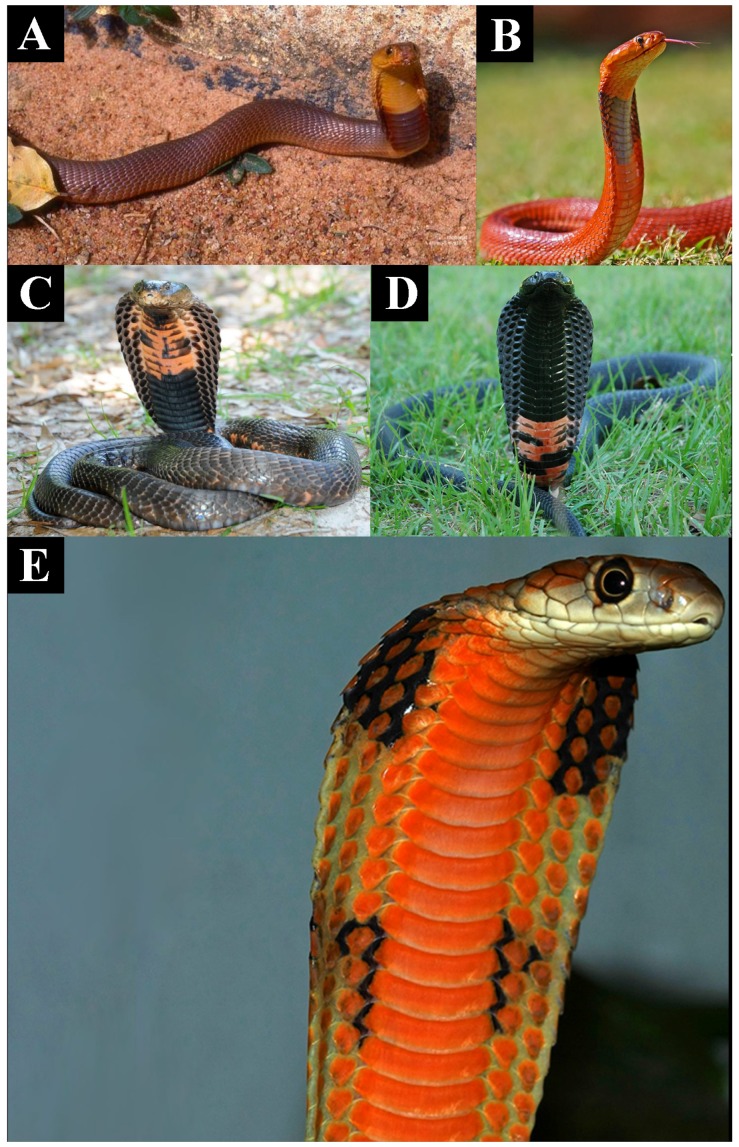
Aposematic hood colouring in the African spitting cobras such as (**A**) *Naja katiensis*; (**B**) *Naja pallida*; (**C**) *Naja mossambica*; (**D**) *Naja nigricollis*; and (**E**) convergently in the adult colouring in the Malaysian population of *Ophiophagus hannah* (the most cytotoxic *O. hannah* population). Photos (**A**) Stephen Spawls; (**B**) Wikimedia Commons; (**C**,**D**) Randy Ciuros; (**E**) Kevin Messenger.

Within the African *Naja* spitting cobras, *N. katiensis* (Figure 6A), which is a relatively dull red, has less cytotoxic venom than the scarlet *N. pallida* (Figure 6B) [32]. While *N. mossambica* (Figure 6C) and *N. nigricollis* (Figure 6D) both exhibit bright red aposematic markings on their necks, there is a notable difference in the location and colour of the markings. *N. nigricincta* represents a aposematically black and white banded derivation within this aposematically red coloured clade (Figure 6C). In both cell types the *N. nigricollis* venom was the most potent, which is congruent with documented effects on human bite victims. Similarly (and apparently convergently), some populations of *O. hannah* (e.g., those from Malaysia) in which adults are golden in colour with subtle reticulated patterns instead of aposematically banded, snakes exhibit vibrant orange aposematic colouring on the front side of the hood analogous to the red markings of the African spitting cobras (Figure 6E). The Malaysian population was also the most cytotoxic of the *Ophiophagus* populations tested (Figure 1 and Supplementary Tables).

**Figure 7:**
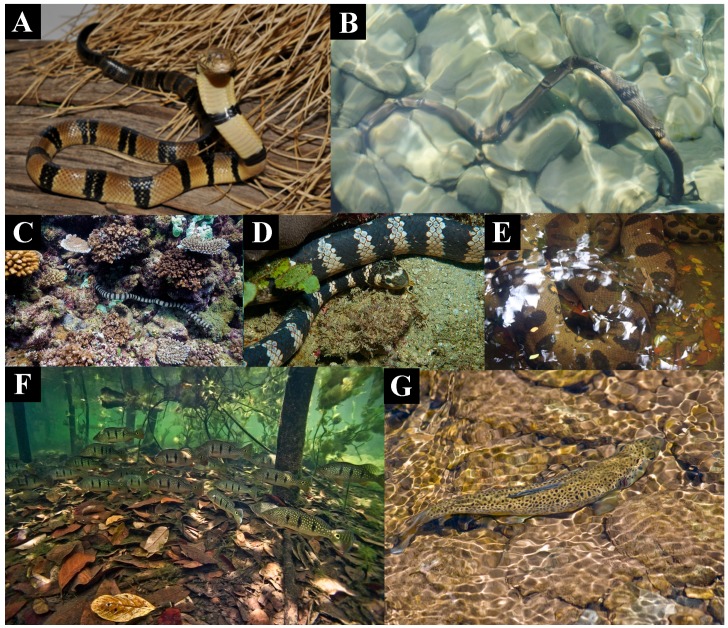
Disruptive camouflage patterning in aquatic snakes (**A**,**B**) *Naja annulata* which also has a secondarily extremely reduced hood and also secondarily lost its cytotoxicity; (**C**) *Laticauda colubrina*; (**D**) *Emydocephalus annulatus*; (**E**) *Eunectes murinus* and fish (**F**) *Cichla orinocensis*; (**G**) *Salmo trutta*. Photos (**A**) Markus Oulehla; (**B**,**D**) Wikimedia Commons; (**C**) Jan Messersmith; (**E**) Rhett A. Butler; (**F**) Ivan Mikolji; (**G**) Phil Skinner.

**Figure 8:**
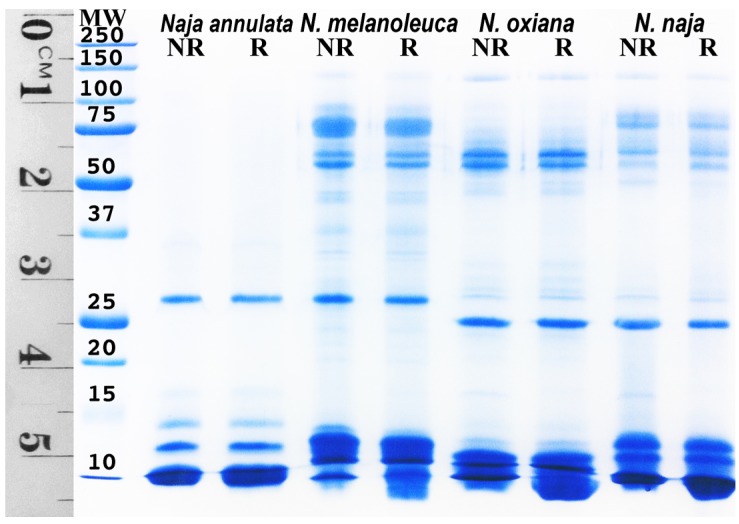
1D SDS-PAGE mini-gel showing the streamlining of the aquatic cobra species *Naja annulata* in comparison to the closest relative *N. melanoleuca*, and the Asian non-spitting species *N. oxiana* and *N. naja.* Running conditions: NR = non-reduced, R = reduced.

**Figure 9:**
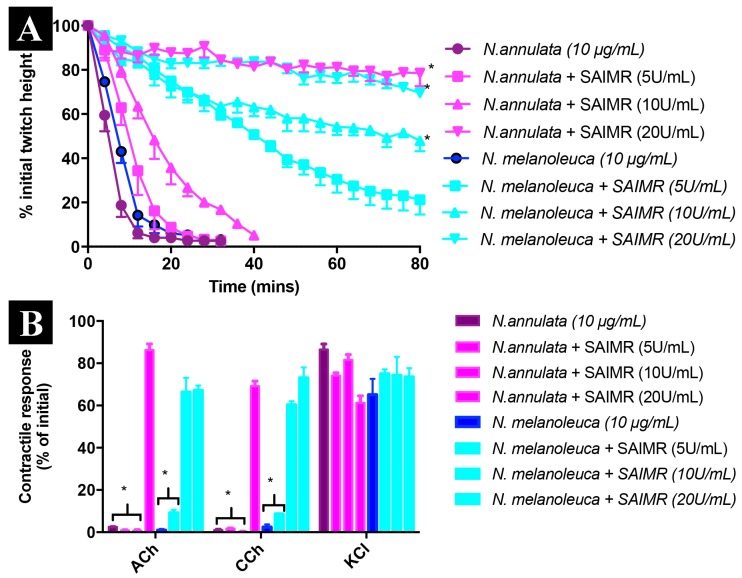
Neurotoxic effects of *Naja annulata* and *Naja melanoleuca* alone (10 µg/mL) and in the presence of SAIMR polyvalent antivenom (SAIMR PAV; 5, 10, 20 U/mL; *n* = 3) on (**A**) indirect twitches (**B**) responses to exogenous agonists the chick biventer cervicis preparation. * *p* < 0.05, significantly different to venom alone.

In contrast to the evolution of aposematic body banding, the banding of the water cobra *N. annulata* likely serves a camouflage purpose in the aquatic niche they occupy, similar to that selected for in other aquatic lineages from sea snakes to fish (Figure 7). As well as for the loss of aposematic hood markings, there would be a selection pressure in this environment for the shortening of the elongate ribs of the hood in order to increase streamlining and allow great neck flexibility for swimming and hunting fish in crevices. In both cell types tested *N. annulata* was significantly less cyototoxic than all other *Naja* (Figure 1 and Supplementary Tables). The venom of this species has also undergone a proteomic streamlining (relative to that of other *Naja—*Figure 8) in a manner analogous to the evolution of simplified venoms in other aquatic elapid snakes—sea kraits and sea snakes [33,34]. The absence of cytotoxins in the venom of this species is thus linked to the behavioural changes associated with occupying a new ecological niche, that of an aquatic snake that spends most if not all of its time in or around a body of water and has undergone a shift to a primarily piscivorous diet. The easy escape mechanism of fleeing into the water [35], an almost unique adaptation within *Naja*, may be the reason for the lack of cytotoxins in the venom of this species. The loss of strong cytotoxic activity and reduction of hooding as a defensive behaviour are both derived character states of *N. annulata* and this shift is reflected in the degree of relative neurotoxicity, with *N. annulata* being more neurotoxic than its closest relative *N. melanoleuca* (Figure 9A) with t_90_s of 10 min and 15 min respectively. The neurotoxic action was mediated by alpha-neurotoxins (Figure 9B), which are retained in *N. melanoleuca.* Thus, as the latter species is used in the antivenom immunizing mixture, cross-reactivity occurred with the *N. annulata* alpha-neurotoxins (Figure 9A).

**Figure 10:**
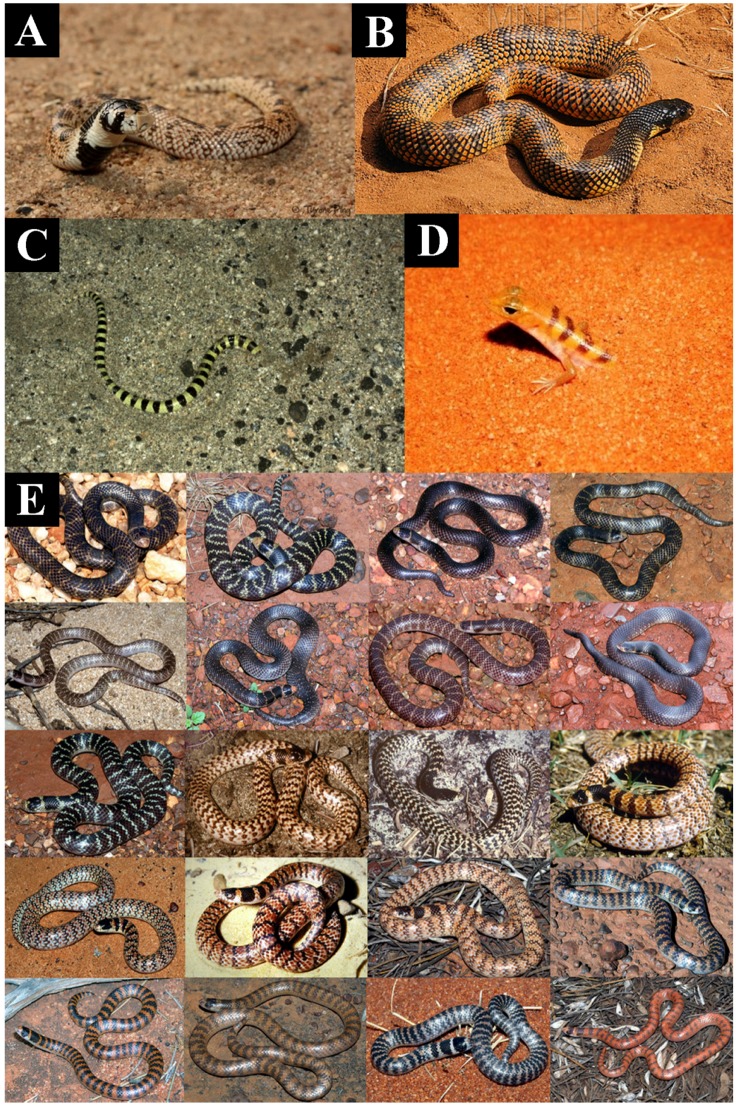
Disruptive camouflage patterning the nocturnal semi-fossorial reptiles (**A**) *Aspidelaps scutatus*; (**B**) *Aspidelaps lubricus*; (**C**) *Chionactis occipitalis*; (**D**) *Eremiascincus fasciolatus*; and (**E**) the hypervariable *Brachyurophis* genus (counting from the left starting with the top row) *B. approximans* (1–9), *B. fasciolatus* (10–15) and *B. semifasciatus* (16–20). Photos (**A**) Tyrone Ping; (**B**) Tony Phelps; (**C**) Richard Cazares; (**D**) Wikimedia Commons; (**E**) Brian Bush.

The banding and body marking of *Aspidelaps* is convergent with that of other nocturnal, semi-fossorial reptiles and likely functions as camouflage, not as an aposematic marker (Figure 10). While *A. lubricus* is significantly less cytotoxic than even *N. haje*, it is still notably more cytotoxic than *A. scutatus* (Figure 1 and Supplementary Tables). Consistent with this, if caught out in the open during the day, the orange and black banding of *A. lubricus* may well serve an ancillary (and perhaps epiphenomenal) aposematic function, just as aposematically banded diurnal species would benefit from the banding as camouflage during nocturnal activity periods. This “dual-purpose” of banding suggests a possible evolutionary mechanism for the origin of aposematic banding in snakes. Extrapolating from these evolutionary patterns and from the low cytotoxicity of the narrow-hooding, semi-fossorial *Aspidelaps* species (Figure 1 and Supplementary Tables), we hypothesise that venom of the burrowing cobra *Naja multifasciata* (not investigated in the present study) is likely to possess reduced cytotoxicity as it also has secondarily reduced its hood and defensive displays parallel to the evolution of a fossorial lifestyle. Similarly, the tree cobra *N. goldii* (not investigated in this study) also has a secondary reduction of hood and defensive displays parallel to the evolution of an arboreal lifestyle and so we likewise hypothesise a reduced cytotoxicity for its venom.

Mapping these changes over the phylogenetic tree allows for a ready visualisation of this complex interplay (Figure 1). It should also be noted that while the evolution of the upright hooding display is convergent between *Hemachatus* *+* *Naja* and *Ophiophagus*, so is the evolution of gross cytotoxicity, with the specific cytotoxin types used differing between these two clades. The *Naja* *+* *Hemachatus* clade utilise derived 3FTx peptides (cytotoxins or “cardiotoxins”) [1], whereas *Ophiophagus* utilises L-amino acid oxidase (LAAO) [31] enzymes in the same functional role. Thus there is convergence between the two groups in upright hooding displays being associated with defensive cytotoxic function, and as they have evolved the function independently the underlying chemical mechanisms are not homologous. *Hemachatus* *+* *Naja* venoms have a high concentration of the cytotoxic 3FTx unique to this clade [36,37,38,39,40,41,42,43,44,45]. Similarly *O. hannah* venoms have the highest concentration of L-amino acid oxidase of any snake venom and also the most derived forms of venom L-amino acid oxidase [23,46,47,48,49,50,51]. Taken together these results are strongly indicative of a co-evolutionary relationship between hooding behaviour and cytotoxic venom.

Cytotoxic 3FTx make very little contribution to the lethal effects of cobra venom, having LD_50_s ranging from 3.8 to 9.7 mg/kg [52,53]. In comparison alpha-neurotoxic 3FTx (also present in the venoms of cobras) have intravenous LD50s of 0.07–0.2 mg/kg [34]. Cytotoxic 3FTx are thus likely to be considerably less effective in prey subjugation than their neurotoxic peptide ancestors. This, along with the demonstration in the present study that cytotoxicity is associated with an obvious defensive adaptation (hooding behaviour), makes a strong case that their evolution has been shaped by the defensive deployment of venom. Similar results are available for the LAAO enzymes responsible for the cytotoxicity of *O. hannah* venom [54], with this enzyme class having an unimpressive LD_50_ of 5 mg/kg [49]. Low toxicity and pain-inducing activities are properties of defensive toxins observed in other lineages [55]. The use of venom in defense by snakes has been a contentious subject, given that loss or reduction of the venom system has been observed in some species subsequent to a transition to defenseless prey [56,57,58,59,60]. It has been suggested, however, that maintenance of the venom system for a predatory function might facilitate defensive deployment [61]. Certainly, all species in the present study utilise their venom in predation, but to our knowledge this is the first study to demonstrate strong evidence of the evolution of certain snake venom components (toxins) driven largely by a defensive function. This is congruent with the streamlining of the venom (Figure 8) and rise in relative neurotoxicity (Figure 9) paralleling the secondary loss of cytotoxicity of in the aquatic *N. annulata*, with these changes accompanied by a loss of the long hood ribs (Figure 7A).

It has long been clear that spitting cobras possess morphological and behavioural traits that evolved for defensive purposes [28], but the relationship between these traits and venom composition has been uncertain. As cytotoxins are the primary toxins implicated in the ocular irritation and damage caused by spat *Naja* venom, our results suggest a clear evolutionary trajectory in which the evolution of hooding as a confrontational defensive behaviour likely led to selection for a damaging, pain-inducing venom as a result of its new major role, which then facilitated the evolution of specialised mechanisms of long-distance venom delivery in three independent lineages. Reliance on bold defensive displays likely increases the frequency with which snake species are forced to deploy their venom defensively, thus driving the selection of venom that causes painful local damage to potential predators. Both the confrontational nature of the hooding display and its upright posture may be crucial in the evolution of spitting behaviour, which could explain why only snakes that adopt this defensive posture have evolved spitting, i.e., it is necessary for a snake to be looking forward and upwards at incoming predators for them to be able to spit venom effectively. As to why spitting behavior has never evolved in *Ophiophagus* despite the potent cytotoxicity providing the selection pressure of venom “worth spitting”, it may be that the very large, globular enzymes underlying this function in *Ophiophagus* venom are less efficiently absorbed in the eyes than are the very small peptides that underpin the cytotoxic function in *Naja*. Thus, it is clear that a strong co-evolutionary relationship exists between three of the character states that define cobras as a group: exceptional hooding displays (which have evolved twice within the clade), spitting behaviour/associated morphology (evolved three times within the clade) and increases in cytotoxic activity linked to the evolution of aposematic markings. Nevertheless, it remains a mystery why hooding (at least to the extent seen in cobras) is relatively rare in snakes and therefore what factors initiate the above scenario for evolution of this defensive suite.

To summarise, in *Naja* there is extensive variation in hooding (Figure 4), aposematic markings (Figure 4, Figure 5 and Figure 6) and camouflage (Figure 7 and Figure 10) and these variables are linked to patterns in relative cytotoxicity (Figure 1 and Supplementary Tables), venom complexity (Figure 8) and/or changes in neurotoxicity (Figure 9). In contrast to the variation within *Naja* a high level of defensive cytotoxicity is preserved across the full range of *Ophiophagus* venoms examined in this study, as are the morphological and behavioural attributes of hooding in this species. It is notable, however, that the Malaysian population with bright orange aposematic hood colouration in adult snakes (Figure 6E) is also the most cytotoxic population. This relative conservation of L-amino acid driven defensive cytotoxicity is in contrast to documented variation among *O. hannah* populations in the relative presence and concentration of toxins used in predation [62]. Thus, as discussed above, this species provides additional evidence that hooding is associated with defensive cytotoxic activity even in the face of substantial variation in the toxins selected for use in predation.

Overall, our results suggest that cytotoxicity evolved first with hooding. Hooding precedes several independent increases in cytotoxicity in the *Naja* + *Hemachatus* clade and evolved concurrently with increased cytotoxicity in *Ophiophagus* (Figure 1 and Supplementary Tables). No spitting accompanies the increase in cytotoxicity in *N. annulifera*. However, spitting is clearly closely associated with a secondary increase in cytotoxicity in *Hemachatus* and African *Naja*. The Asian *Naja* could either have evolved spitting at the same time as increased cytotoxicity or possibly afterwards, depending on whether weakly spitting *N. atra* and *N. kauothia* specimens are deemed informative. The inverse relationship between classes of aposematic marking (ornate hood patterns or flashes of red on the hood versus bold body banding) is an intriguing aspect, but in either case these aposematic markings are linked to further rises in cytotoxicity.

Given the significant human impact of the cytotoxic venoms of *Naja* sp. in both sub-Saharan Africa and southern Asia, the results of this study are also evidence of the mutually enlightening relationship between evolutionary toxinology and clinical toxinology. Understanding the evolutionary selection pressures resulting in the evolution of cytotoxic venom can help us understand the association between snake behaviour and the impact of snakebite, and could also be utilised to develop next-generation snakebite therapeutics, which are particularly desirable for the treatment of local damage inflicted by cytotoxins, as this common, high-impact, result of envenomation is poorly treated by currently available antivenoms [63]. In addition, the investigation of novel cytotoxins may provide lead compounds for the use in anticancer drug design and development.

## 3. Materials and Methods

### 3.1. Species Studied

- Non-hooding species
  - Bungarus fasciatus
  - Dendroaspis polylepis
  - Elapsoidea boulengerii, Elapsoidea sundevali longicauda, Elapsoidea sundevali sundevali
  - Walterinnesia aegyptia
  - Naja annulata
- Narrow hooding species
  - *Aspidelaps lubricus, Aspidelaps scutatus* (nar)
- Hooding species
  - Hemachatus haemachatus
  - African *Naja*
    - Non-spitters = *N. annulata*, *N. annulifera*, *N. haje*, *N. melanoleuca*, *N. nivea*
    - Spitters = *N. mossambica*, *N. nigricollis*, *N. pallida*
  - Asian *Naja*
    - Non-spitters = *N. atra*, *N. kaouthia*, *N. naja*, *N. oxiana* (Note that *N. atra* and *N. kaouthia* are slightly ambiguous as they are not considered as true spitting cobras, and lack the highly specialised adaptations of the other species I this category, but have been documented to spit significantly under strong duress [27,28]
    - Spitters = *N. philippinensis*, *N. siamensis*, *N. sumatrana*
  - *Ophiophagus hannah* clade (Four geographically different samples from *O. hannah* from Cambodia, Malaysia, Thailand, East Java).

Hooding and spitting were categorized based on experience with these animals combined with literature sources. For the comparative analyses described below, two separate datasets were used when investigating either hooding or spitting. One wherein ambiguous species were considered to lack the trait and another wherein ambiguous species were considered to have the trait. Ambiguous species for hooding were *Aspidelaps lubricus* and *A. scutatus* and those for spitting were *Naja atra* and *N. kaouthia*, such that the number of species affected by alternative coding was small but it still allowed us to ensure robustness of results to differing interpretations.

### 3.2. Venom Preparation

Venoms were obtained in lypholized form and prepared by resuspension in deionized water (dH_2_O), centrifugation at 4000 RCF, and supernatant passed through a 0.45 µm Millipore (Brisbane, QLD, Australia) syringe driven filter unit. Protein concentrations were determined using a NanoDrop 2000 UV-Vis Spectrophotometer (Thermofisher, Sydney, NSW, Australia) at an absorbance of 280 nm.

### 3.3. Cell Lines and Cell Culture

The effect of each snake venom was assessed on human neonatal foreskin fibroblast (NFF) and malignant melanoma (MM96L) cell lines, supplied by QIMR Berghofer Medical Research institute. Venom mediated cytotoxicity is often responsible for the degradation and destruction of skin and connective tissue, therefore the chosen cell lines were deemed appropriate. Cell lines were maintained in RPMI medium supplemented with 1% penicillin-streptomycin and foetal calf serum (FCS), 10% FCS for NFF and 5% FCS for MM96L. Cells were split 24 h prior to the experiment (for up to 25 passages for MM96L and 10 passages for NFF) using 0.25% trypsin, and seeded in 96-well flat bottom plates at a density of 5000 and 2500 cells/well for NFF and MM96L cells, respectively. Plates were incubated overnight at 37 °C in a 5% CO_2_-95% humidified environment prior to treatment.

### 3.4. MTT Assays

Cell viability was evaluated using colorimetric MTT (Thiazolyl Blue Tetrazolium Bromide; Sigma-Aldrich M5655, Sydney, NSW, Australia) assays. Venom was added to cells at 5 µg, 1 µg, 0.5 µg, and 0.1 µg protein amounts and followed by a 48-h incubation period. MTT was added at a concentration of 5 mg/mL per well. An amount of 0.1% sodium dodecyl sulphate (SDS) was used as a positive control to achieve 100% toxicity, and the protocol was followed according to the manufacturer’s description. The absorbance was read at 570 nm on the PowerWave XS2 plate-reader (Bio-Tek Instruments, Winooski, VT, USA), using Gen5 software. Three independent experiments were conducted with a minimum of three replicates per treatment. Cell viability readings were normalized as a percent of untreated control cells, and viability expressed as a percentage of toxicity ± standard error of the mean (SEM). The relationship between venom dose and cytotoxic response was calculated via area under the curve (AUC) analysis, using GraphPad Prism 7 (GraphPad Software, Inc., La Jolla, CA, USA) (Supplementary Tables).

### 3.5. Phylogenetic Comparative Analyses

A phylogeny was assembled using [11] as a starting point as this is currently the most comprehensive time-calibrated phylogeny available for elapids. This tree included all species within the current dataset except *Aspidelaps lubricus*, *Elapsoidea boulengeri* (and the two *E. sundevallii* ssp.), *Naja oxiana*, *N. philippinensis*, and the localities of *N. naja* and *Ophiophagus hannah*. Consequently, these species were added to the tree (by directly manipulating the newick-formatted file) at positions which were deemed plausible to give a final working phylogeny. *A. lubricus* and *E. boulengeri* were added as sister species to their congeners with a divergence of 5 mya as this is approximately representative of the divergence of many sister species in this tree. For *E. sundevallii* ssp. and *O. hannah* localities, we created a split and a polytomy (respectively) at 2.5 mya for the same reasons (half the 5 mya to reflect the smaller divergence within species). *N. philippinensis* was included as a polytomy with *N. sumatrana* and *N. siamensis* because the relationships in this clade are currently very uncertain, however due to the general similarity of *N. oxiana* and *N. naja*, we added the former as a sister species to the latter at the midpoint of the divergence between *N. naja* and *N. atra* (sister groups before the inclusion of *N. oxiana*). For the two *N. naja* populations, we added those as a split diverging midway from the divergence from *N. oxiana*. The resulting phylogeny is shown in Figure 1 and was used for all further analyses conducted in R v3.2.5 [64] using the ape package [65] plus others introduced for particular analyses.

First, the phylogenetic signal of all traits used in the following analyses was assessed to examine to what extent variation in the traits reflected evolutionary history. For binary traits (hooding and spitting), we estimated Fritz and Purvis’ D [66] in the R package caper [67], testing for significant signal using 5000 permutations. For continuous traits (cytotoxicity measured as AUC for each cell line), we estimated Blomberg et al.’s K [68] in the R package phytools [69], and similarly tested for significance using 5000 simulations. In all cases, detectable phylogenetic signal was found (*p* < 0.0002 for spitting, however ambiguous species were coded and also for hooding when ambiguous species coded as not hooded; *p* = 0.001 for hooding when ambiguous species were coded as having hoods and also for cytotoxicity to the NFF cell line; *p* = 0.0002 for cytotoxicity to the MM96L cell line), emphasizing the need to analyze the data in a comparative framework.

Ancestral states were estimated and reconstructed over the tree in order to investigate the evolutionary history of the traits and consequently their relation to one another over time. For binary traits (spitting and hooding), we used the rerooting method [70] in phytools [69] as this method can handle polytomies when reconstructing categorical traits. The underlying model used was an equal rates model for both traits, chosen after log-likelihood comparison with a different rates model. The continuous traits (cytotoxicity on each cell line measured as AUC) were reconstructed by maximum likelihood in the contMap function in phytools [69].

To compare the strength of convergence in cytotoxicity in association with hooding vs. spitting, we calculated the Wheatsheaf index [29] for cytotoxicity as a two-dimensional trait (i.e., combining both cell lines) with either hooding or spitting as the focal trait. We were mainly interested in comparing the index between the two categorical traits but also tested for a signal of extremely strong convergence using 1000 bootstrap replicates to generate a null distribution. These analyses were conducted in the R package windex [71].

As a final estimate of the relative importance of hooding and spitting in the evolution of cytotoxicity, we fit a series of pGLS models in caper [67]. The same strategy was used for each of four datasets: the two coding versions for ambiguous species and AUC for the two cell lines. For each dataset the model set consisted of four models with cytotoxicity as the response variable and either hooding, spitting, both, or neither (intercept-only) as explanatory variables. Models were then compared with Akaike information criterion (AIC) and where the best model was not the null (intercept-only) model the output was checked to ensure that the explanatory variable had a significant effect before being finally accepted as the best model.

### 3.6. SDS-PAGE

One-dimensional sodium dodecyl sulfate polyacrylamide gel electrophoresis (1D SDS- PAGE) was performed as per previously described methods [72] to compare *N. annulata*, *N. melanoleuca*, *N. oxiana* and *N. naja* venoms.

### 3.7. Neurotoxicity Assays

The in vitro neurotoxicity of venoms was tested using the chick biventer cervices nerve-muscle preparation as described previously [73]. This procedure was approved by the Monash Animal Research Platform (MARP) Animal Ethics Committee, Monash University, Australia MARP/2014/97 (approved in December 2014).

## Supplementary Materials

The following are available online at www.mdpi.com/2072-6651/9/3/103/s1, Table S1: NFF venom cytotoxicity, Table S2: MM96L venom cytotoxicity.
