# Supplementary material for: How the Cobra Got Its Flesh-Eating Venom: Cytotoxicity as a Defensive Innovation and Its Co-Evolution with Hooding, Aposematic Marking, and Spitting
Source: Toxins (Basel). 2017 Mar 13;9(3):103. doi: 10.3390/toxins9030103 (PMC5371858; doi:10.3390/toxins9030103)
Supplement: Supplementary file 1 [file toxins-09-00103-s001.pdf]

Supplementary Materials: How the Cobra Got Its Flesh-Eating Venom: Cytotoxicity as a Defensive Innovation and Its Co-Evolution with Hooding, Aposematic Marking, and Spitting

Nadya Panagides, Timothy N. W. Jackson, Maria P. Ikonomopoulou, Kevin Arbuckle, Rudolf Pretzler, Daryl C. Yang, Syed A. Ali, Ivan Koludarov, James Dobson, Brittany Sanker, Angelique Asselin, Renan C. Santana, Iwan Hendrikx, Harald van der Ploeg, Jeremie Tai-A-Pin, Romilly van den Bergh, Harald M.I. Kerkkamp, Freek J. Vonk, Arno Naude, Morné A. Strydom, Louis Jacobsz, Nathan Dunstan, Marc Jaeger, Wayne C. Hodgson, John Miles and Bryan G. Fry

Table S1. NFF venom cytotoxicity (%) ± SEM.

Species	Origin	5 µg	1 µg	0.5 µg	0.1 µg	AUC
A. lubricus	Africa	99.93 ± 0.07	39.50 ± 7.67	24.07 ± 2.08	6.26 ± 5.88	62.19
A. scutatus	Africa	99.46 ± 0.13	35.25 ± 0.75	0.38 ± 0.2	2.37 ± 2.37	53.24
B. fasciatus	Asia	99.16 ± 0.15	28.63 ± 2.42	25.84 ± 2.52	14.50 ± 6.60	49.99
D. polylepis	Africa	29.02 ± 3.36	24.48 ± 2.26	19.80 ± 4.30	10.01 ± 2.63	98.76
E. boulengeri	Asia	100.00	19.45 ± 4.22	14.73 ± 1.78	2.59 ± 1.01	49.83
E. s. longicauda	Asia	99.68 ± 0.12	12.29 ± 0.96	5.09 ± 1.76	4.66 ± 2.44	39.19
E. s. sundevalli	Asia	99.85 ± 0.11	4.15 ± 2.30	2.94 ± 1.88	5.96 ± 2.68	36.66
H. haemachatus	Africa	98.84 ± 0.21	100.00	100.00	0.00	134.5
N. annulata	Africa	34.95 ± 4.79	12.51 ± 1.79	13.97 ± 3.4	7.98 ± 2.03	54.45
N. annulifera	Africa	99.81 ± 0.12	98.90 ± 0.44	70.30 ± 6.42	0.00	119.7
N. atra	Asia	99.08 ± 0.13	97.83 ± 0.37	98.16 ± 0.22	22.71 ± 3.72	133.5
N. haje	Africa	99.91 ± 0.08	51.41 ± 9.37	7.58 ± 3.45	5.70 3.62	60.21
N. kaouthia	Asia	99.46 ± 0.18	99.96 ± 0.04	98.38 ± 0.74	0.00	134
N. melanoleuca	Africa	99.84 ± 0.13	58.33 ± 4.76	0.00	0.64 ± 0.64	64.38
N. mossambica	Africa	98.95 ± 0.19	96.57 ± 0.81	33.81 ± 8.54	0.00	100.8
N. naja	Asia (India)	97.92 ± 0.4	99.89 ± 0.11	98.42 ± 0.26	5.57 ± 0.64	133.4
N. naja	Asia (Pakistan)	98.87 ± 0.17	100.00	99.19 ± 0.48	4.71 ± 2.45	134.1
N. nigricollis	Africa	99.03 ± 0.3	99.96 ± 0.3	92.59 ± 3.08	0.00	130.9
N. nivea	Africa	99.27 ± 0.13	86.53 ± 4.69	44.77 ± 3.13	4.81 ± 2.31	99.35
N. oxiana	Asia	100.00	98.50 ± 0.6	41.83 ± 6.72	5.77 ± 5.58	103.3
N. pallida	Africa	97.63 ± 0.25	98.59 ± 0.65	32.73 ± 10.21	0.00	101.2
N. philippinensis	Asia	99.49 ± 0.13	90.62 ± 0.81	0.00	0.00	80.49
N. siamensis	Asia	99.70 ± 0.07	97.92 ± 0.1	94.09 ± 1.71	64.33 ± 1.09	124.5
N. sumatrana	Asia	99.27 ± 0.19	98.53 ± 0.31	97.72 ± 0.63	14.69 ± 5.26	133.6
O. hannah	Asia (Cambodia)	100.00	100.00	99.49 ± 0.51	26.52 ± 2.70	134.6
O. hannah	Asia (Java)	99.97 ± 0.03	100.00	100.00	9.22 ± 4.76	134.9
O. hannah	Asia (Malaysia)	100.00	100.00	99.9 ± 0.1	42.10 ± 6.3	134.9
O. hannah	Asia (Thailand)	100.00	100.00	100.00	24.00 ± 1.83	134.9
W. aegyptia	Africa	100.00	98.64 ± 0.03	40.54 ± 9.32	1.45 ± 1.13	104.1

Table S2. MM96L venom cytotoxicity (%) ± SEM.

Species	Origin	5 µg	1 µg	0.5 µg	0.1 µg	AUC
A. lubricus	Africa	99.17 ± 0.44	33.39 ± 2.12	0.34 ± 0.34	0.00	51.95
A. scutatus	Africa	100.00	27.93 ± 2.69	0.07 ± 0.07	0.00	48.95
B. fasciatus	Asia	98.77 ± 0.16	8.54 ± 4.27	4.81 ± 2.44	0.00	41.71
D. polylepis	Africa	18.60 ± 3.59	7.06 ± 4.28	0.05 ± 0.05	0.26 ± 0.26	54.24
E. boulengeri	Asia	78.22 ± 6.47	61.74 ± 4.07	59.11 ± 4.79	15.38 ± 1.49	106.6
E. s. longicauda	Asia	50.53 ± 11.64	32.29 ± 4.10	21.69 ± 5.55	6.49 ± 1.95	81.5
E. s. sundevalli	Asia	67.08 ± 10.75	60.28 ± 3.84	39.56 ± 6.97	9.12 ± 1.66	105.3
H. haemachatus	Africa	99.69 ± 0.12	100.00	100.00	16.02 ± 3.29	134.8
N. annulata	Africa	6.71 ± 2.19	0.00	3.34 ± 2.36	1.79 ± 1.79	69.2
N. annulifera	Africa	100.00	99.94 ± 0.06	76.75 ± 4.88	0.00	123.3
N. atra	Asia	99.94 ± 0.03	99.97 ± 0.03	100.00	19.26 ± 1.15	134.9
N. haje	Africa	100.00	70.57 ± 4.09	6.62 ± 3.49	4.80 ± 1.54	70.45
N. kaouthia	Asia	100.00	100.00	100.00	3.12 ± 0.49	134.9
N. melanoleuca	Africa	100.00	89.70 ± 4.27	3.46 ± 1.66	0.00	81.53
N. mossambica	Africa	99.91 ± 0.08	100.00	99.65 ± 0.32	2.43 ± 2.43	134.7
N. naja	Asia (India)	99.69 ± 0.16	100.00	100.00	22.43 ± 3.5	134.8
N. naja	Asia (Pakistan)	98.77 ± 0.16	100.00	100.00	15.59 ± 1.87	134.4
N. nigricollis	Africa	99.95 ± 0.05	100.00	100.00	31.79 ± 5.79	134.9
N. nivea	Africa	99.97 ± 0.03	80.01 ± 5.87	24.09 ± 11.81	1.04 ± 1.04	86.51
N. oxiana	Asia	99.95 ± 0.05	100.00	79.70 ± 7.14	9.37 ± 4.70	123.7
N. pallida	Africa	99.26 ± 0.16	100.00	94.93 ± 5.07	3.57 ± 2.11	132.1
N. philippinensis	Asia	100.00	99.97 ± 0.03	29.05 ± 7.64	0.00	99.46
N. siamensis	Asia	100.00	100.00	99.98 ± 0.02	84.74 ± 3.17	134.9
N. sumatrana	Asia	99.95 ± 0.05	100.00	99.65 ± 0.35	3.48 ± 1.04	134.8
O. hannah	Asia (Cambodia)	100.00	100.00	61.19 ± 8.20	2.38 ± 1.19	115.1
O. hannah	Asia (Java)	99.31 ± 0.61	86.12 ± 3.02	62.73 ± 4.46	18.64 ± 7.21	104.1
O. hannah	Asia (Malaysia)	100.00	100.00	86.78 ± 7.13	12.18 ± 2.24	127.4
O. hannah	Asia (Thailand)	100.00	95.88 ± 2.13	75.26 ± 6.44	7.75 ± 4.63	119.3
W. aegyptia	Africa	60.64 ± 1.75	7.05 ± 1.75	0.00	0.00	40.77
